# Tuberculosis Laboratory Diagnosis Quality Assurance among Public Health Facilities in West Amhara Region, Ethiopia

**DOI:** 10.1371/journal.pone.0138488

**Published:** 2015-09-16

**Authors:** Melashu Balew Shiferaw, Hiwot Amare Hailu, Abebe Alemu Fola, Mulatu Melese Derebe, Aimro Tadese Kebede, Abayneh Admas Kebede, Manamnot Agegne Emiru, Zelalem Dessie Gelaw

**Affiliations:** 1 Bahir Dar Regional Health Research Laboratory Center, Bahir Dar, Ethiopia; 2 Walter and Eliza Hall institute of medical research, Melbourne, Australia; 3 Amhara Regional State Health Bureau, Bahir Dar, Ethiopia; The Foundation for Medical Research, INDIA

## Abstract

**Introduction:**

Reliable smear microscopy is an important component of Directly Observed Treatment Scheme (DOTS) strategy for TB control program in countries with limited resources. Despite external quality assessment is established in Ethiopia, there is lower TB detection rate (48%) in Amhara region compared to the World Health Organization (WHO) estimate (70%). This highlights the quality of smear microscopy needs to be evaluated. Therefore, the aim of this study was to assess the quality of sputum smear microscopy performance among health center laboratories in West Amhara region, Ethiopia.

**Materials and Methods:**

A cross sectional study was conducted from July 08, 2013 to July 07, 2014. Data were collected from 201 public health center laboratories using a structured questionnaire. Slides were collected based on Lot Quality Assurance Sampling (LQAS) method and rechecked blindly by trained laboratory technologists. The data were entered into EPI info V.7 and smear quality indicators and AFB results were analyzed by SPSS version 20.

**Results:**

Among 201 laboratories enrolled in this study, 47 (23.4%) laboratories had major errors. Forty one (20.4%) laboratories had a total of 67 false negative and 29 (14.4%) laboratories had a total of 68 false positive results. Specimen quality, smear thickness and evenness were found poor in 134 (66.7%), 133 (66.2%) and 126 (62.7%) laboratories, respectively. Unavailability of microscope lens cleaning solution (AOR: 2.90; 95% CI: 1.25–6.75; P: 0.013) and dirty smears (AOR: 2.65; 95% CI: 1.14–6.18; P: 0.024) were correlated with false negative results whereas no previous EQA participation (AOR: 3.43; 95% CI: 1. 39-8.45; P: 0.007) was associated with false positive results.

**Conclusion:**

The performance of health facilities for sputum smear microscopy was relatively poor in West Amhara region. Hence, strengthening the EQA program and technical support on sputum smear microscopy are recommended to ensure quality tuberculosis diagnostic service.

## Introduction

Tuberculosis (TB) still remains a major public health problem that ranks as the second leading cause of death from an infectious disease worldwide. Ethiopia is among the 22 high TB and the 27 high Multidrug Resistant (MDR) TB burden countries. In Ethiopia, according to the 2010/11 national TB survey, the prevalence of smear positive TB among adults was 108 per 100,000 populations [1, 2].

Sputum smear microscopy is an important component of Directly Observed Treatment Scheme (DOTS) strategy. It is recommended for detection of infectious TB cases and monitoring of treatment progress in countries with limited resources [3]. However, the quality of the TB diagnostic service has a major influence on the success of the TB control program since false microscopy results could lead to failure in detecting TB patients, unnecessary treatment of non cases and development of MDR-TB [4].

Reliable laboratory service provides results that are consistently accurate. These demands can be met only through commitment to quality assurance. External Quality Assessment (EQA) is a key component of quality assurance program to ensure the quality of sputum smear microscopy. Onsite evaluation, panel testing and blinded rechecking are the methods to be followed for EQA program [5]. In Ethiopia, quality assurance of sputum smear microscopy is adopted to implement at all public health laboratories to improve and sustain the quality of the national TB Program [6].

Although there has been established sputum smear microscopy EQA network, unpublished reports showed lower TB detection rate (48%) in Amhara region [7] compared to the WHO estimate (70%) [5]. This highlights the need to check the quality of sputum smear microscopy. Therefore, this study was aimed to evaluate the current quality of sputum smear microscopy among public health center laboratories in West Amhara region, Ethiopia

## Materials and Methods

### Study design, period and area

A cross sectional study was conducted in public health center laboratories of West Amhara region from 08 July 2013–07 July 2014. The region has five zones and two city administrations that have a total population of 11240275 [7]. The EQA program of TB microscopy is coordinated by Bahir Dar Regional Health Research Laboratory Center in West Amhara region. The program has been decentralized to 26 EQA centers since 2012. These centers implement EQA activities from health facilities in their catchment area through quarterly blind rechecking, and semiannual onsite evaluation and panel testing in the region. Ziehl-Neelsen and/ or Florescent microscopy methods are used to diagnose TB. There are 8 hospitals and 450 health Centers providing sputum smear microscopy diagnostic services in West Amhara region [7].

### Study subjects, sample size and sampling method

Laboratories that have been participated in EQA program, kept all Acid Fast Bacilli (AFB) slides for blind rechecking and reported positive slides based on WHO grading system were enrolled in this study. However, laboratories conducting Fluorescent Microscopy (FM) for pulmonary TB diagnosis were excluded. Major errors were reported in 61.5% of laboratories in Tanzania [8]. A total of 450 laboratories were providing AFB smear microscopy in West Amhara region. Hence, the sample size at 95% confidence interval, 5% marginal error, 450 total population and 61.5% prevalence was 201 as calculated using EPI info v.7. Simple random sampling technique was used to select these 201 laboratories among 450 health center laboratories.

### Data collection procedure and quality control

Trained Woreda health office TB officers and laboratory personnel collected the data using a pretested structured questionnaire. AFB slides were collected based on the LQAS method at 95% CI, zero acceptance number, 100% specificity and 80% sensitivity following the national EQA guideline [6]. Collected slides were transported to the 26 EQA centers. Trained first controllers rechecked the slides blindly at each EQA center. Discordant results between the peripheral laboratory and the first controllers were resolved by senior second controllers.

Error types were interpreted as High False Negative (HFN): misread a 1+ to 3+ positive smear as negative; High False Positive (HFP): misread a negative smear as 1+ to 3+ positive; Low False Positive (LFP): misread a negative smear as scanty (1–9 AFB/100 fields) positive; Low False Negative (LFN): misread a scanty (1–9 AFB/100 fields) positive smear as negative; Quantification Error (QE): difference of more than one grade in reading a positive slide between examinee and controller; major error: smear result with HFN or HFP; minor error: smear result with LFN or LFP or QE [4–6].

Good performance of sputum smear microscopy was taken when the laboratories had no major errors [4]. Specimen quality, smear staining, size, thickness, evenness and cleaning were used as smear quality indicators. Laboratories that scored 80% and above in smear quality indicators were taken as good smear performers [6].

### Data processing and analysis

Data were entered into EPI info v.7 and were analyzed by SPSS version 20 software. Smear quality indicators and AFB results were calculated. Each laboratory was evaluated for major errors, minor errors, and false negative and false positive results. Variables having P value ≤ 0.2 in bivariate analysis were entered into multivariate analysis to manage confounder variables. Significant association was set at P value < 0.05.

### Ethical consideration

Ethical clearance was secured from the Amhara Regional Health Bureau Ethical Review Committee. Official permission was obtained from the Bahir Dar Regional Health Research Laboratory Center and from the head of each public health centers. Results were kept confidential and communicated to the respective laboratories.

## Results

### General characteristics

A total of 201 laboratories were enrolled in this study. Twenty five (12.4%) laboratories had no trained laboratory personnel. Main electric power supply and running water were available in 114 (56.7%) and 37 (18.4%) laboratories, respectively. Three (1.5%) laboratories had separate TB laboratory room. Microscopes were cleaned by recommended lens tissue and cleaning solution in 61 (35.7%) laboratories. Although 157 (78.1%) laboratories had participated in EQA program, 21 (13.4%) didn’t receive feedback reports (Table 1).

**Table 1:** General characteristics of Laboratories in West Amhara region, 2014.

Characteristics		Frequency	%
**Separate room for TB**	No	198	98.5
	Yes	3	1.5
**Separate microscope for TB**	No	167	83.1
	Yes	34	16.9
**Running water in the laboratory**	No	164	81.6
	Yes	37	18.4
**Microscope light source**	Mirror	37	18.4
	Main power	114	56.7
	Solar	50	24.9
**Preventive maintenance**	No	30	14.9
	Yes	171	85.1
**Clean microscope by**	DEE with lens tissue	61	35.7
	70% alcohol with lens tissue	39	22.8
	70% alcohol with cotton/gauze	71	41.5
**Participated in EQA program**	No	44	21.9
	Yes	157	78.1
**Received EQA feedback**	No	21	13.4
	Yes	136	86.6
**Laboratory personnel**	Not trained	25	12.4
	At least on trained	52	25.9
	All trained	124	61.7

TB: tuberculosis; EQA: External Quality Assessment; DEE: Diethyl Ether.

### Reagents and supplies

Shortage of AFB reagents was found in 83 (41.3%) laboratories. Majority of the laboratories (167 [83.1%]) practiced internal quality control for new AFB reagents and routinely in a weekly manner via known positive and negative control smears. AFB reagents had no clear labeling (reagent name, preparation date and expiry date) at 22 (10.9%) laboratories. More than half, 139 (69.2%), laboratories did not filter 1% carbol fuchsin during staining of AFB smears. Microscope lens cleaning solution, filter paper and lens tissue were not found in 127 (63.2%), 92 (45.8%) and 89 (44.3%) laboratories, respectively.

### Annual negative slide volume and slide positivity rate

Majority of the laboratories, 167 (83.1%), had low Slide Positivity Rate (SPR) of AFB slides including 19 (9.5%) laboratories with SPR of 0%. Ninety five (47.3%) laboratories had low Annual Negative Slide Volume (ANSV) (< 301 slides per year). Both low SPR and low ANSV were found in 80 (39.8%) laboratories (Table 2).

**Table 2:** ANSV and SPR of smear microscopy in West Amhara region, 2014.

	SPR <5%	SPR = 5%-10%	SPR>10%	Total
**ANSV**	N (%)	N (%)	N (%)	N (%)
**<301 slides**	80 (39.8)	11 (5.5)	4 (2.0)	95 (47.3)
**301–500 slides**	61 (30.3)	12 (6.0)	2 (1.0)	75 (37.3)
**501–1000 slides**	23 (11.4)	4 (2.0)	1 (0.5)	28 (13.9)
**>1000 slides**	3 (1.5)	0 (0.0)	0 (0.0)	3 (1.5)
**Total**	167 (83.1)	27 (13.4)	7 (3.5)	201 (100.0)

ANSV: Annual Negative Slide Volume; SPR: Slide Positivity Rate; N: number.

### Specimen, staining and smear quality of AFB slides

**Fig 1:**
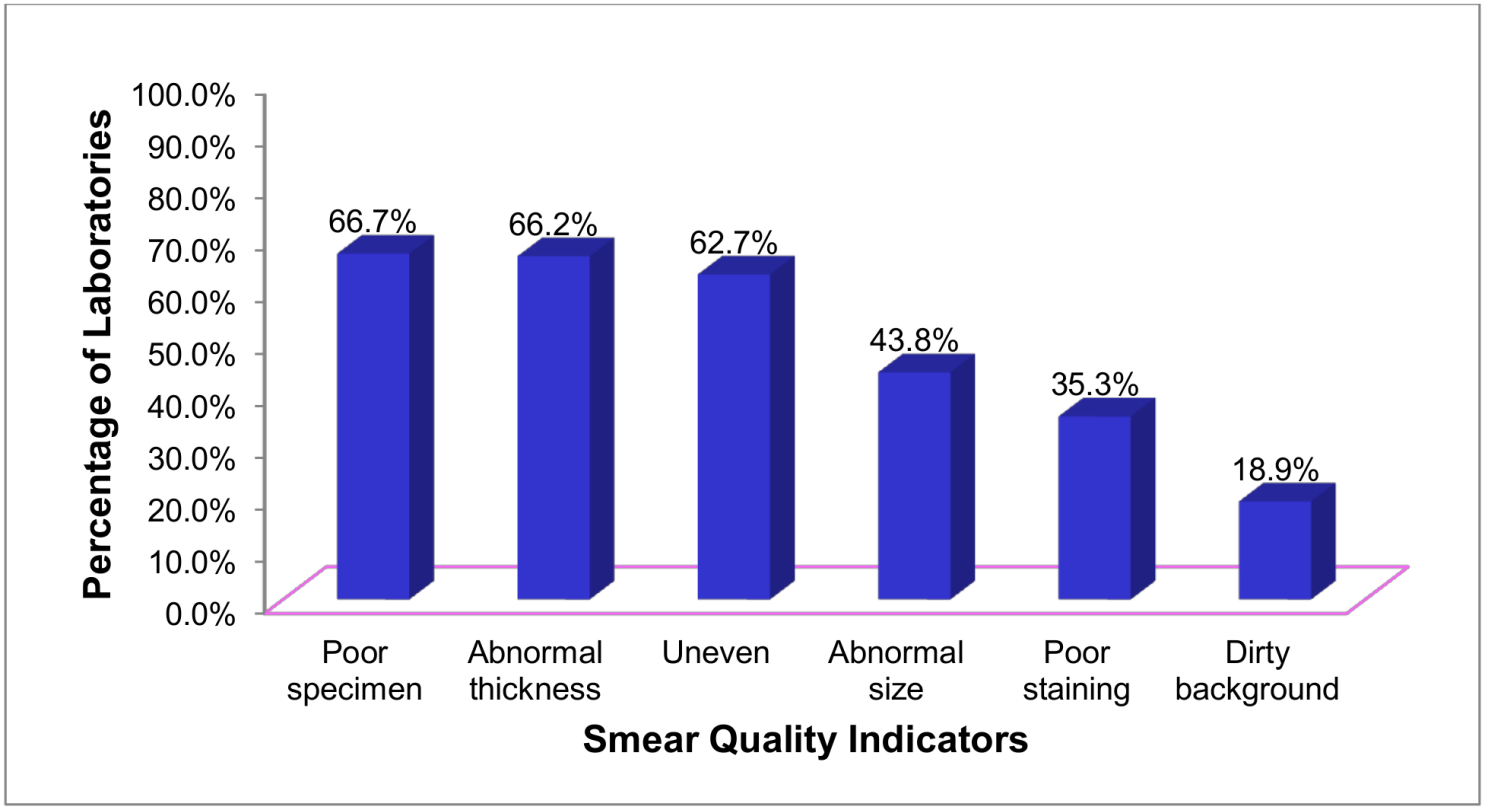
Performance of specimen quality, staining and smearing techniques among laboratories in West Amhara Region, 2014.

Of the 39725 slides rechecked blindly, 35110 (88.4%) slides had clean background, 33222 (83.6%) good staining and 31666 (79.7%) normal size. Good specimen quality, normal thickness and good evenness accounted 29138 (73.3%), 28673 (72.2%) and 28377 (71.4%), respectively. However, 183 (88.4%) laboratories did not fulfill the overall six indicators of smearing quality. Specimen quality, smear thickness and evenness were found poor in 134 (66.7%), 133 (66.2%) and 126 (62.7%) laboratories, respectively (Fig 1).

### Sputum smears microscopy performance

**Fig 2:**
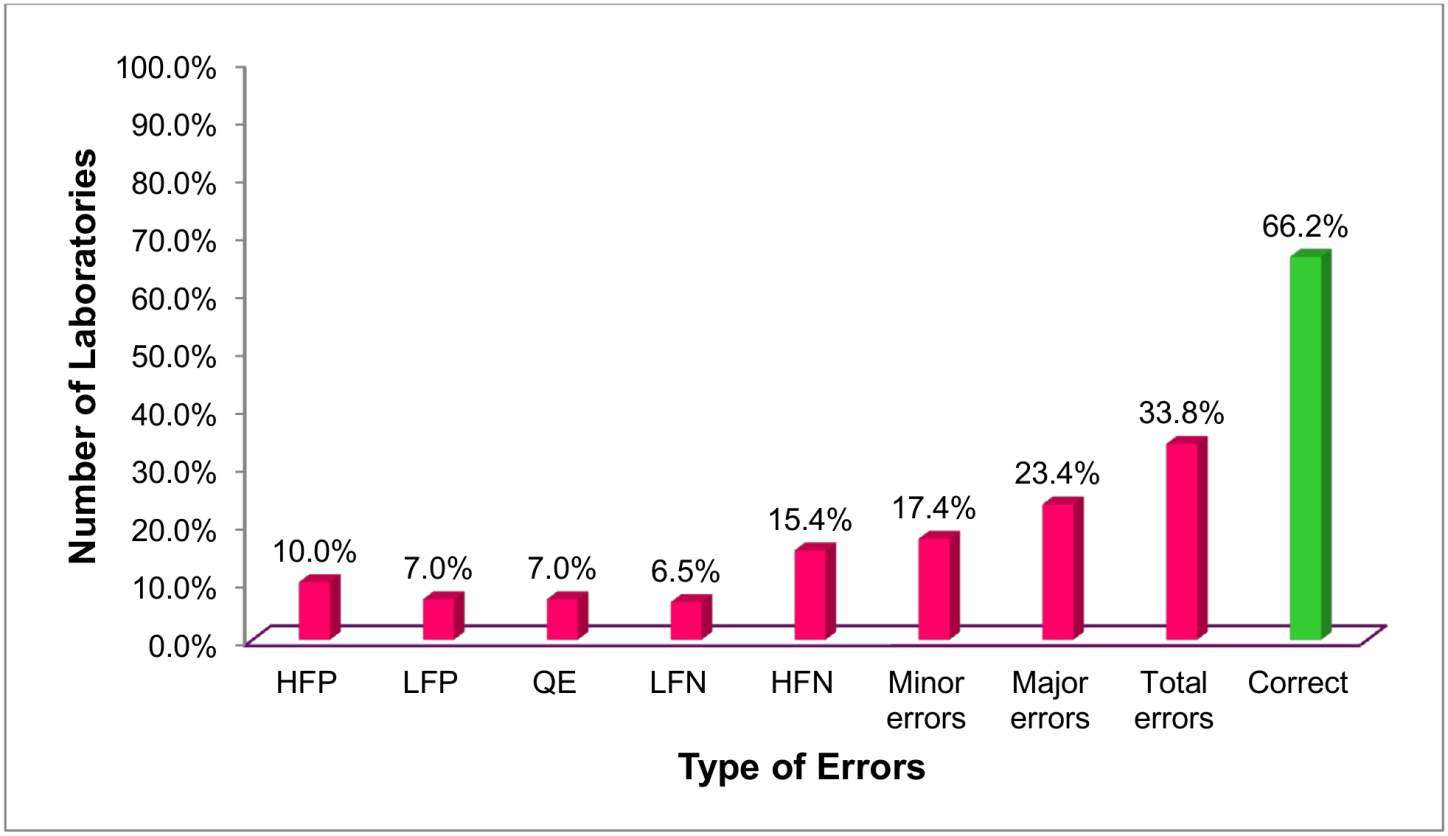
Performance of sputum smears microscopy in West Amhara Region, 2014. HFN: High False Negative; HFP: High False Positive; LFP: Low False Positive; LFN: Low False Negative; QE: Quantification Error.

Of the 201 laboratories, 68 (33.8%) had at least one microscopy error (a total of 68 false positive, 67 false negative and 26 quantification errors). At least one major error was found from 47 (23.4%) laboratories. Forty one (20.4%) laboratories had false negative and 29 (14.4%) laboratories had false positive results (Fig 2).

### Factors for false negatives and false positives

Unavailability of microscope lens cleaning solution and dirty smears were correlated with false negative results. Those laboratories with no lens cleaning solution had significantly higher (AOR: 2.90; 95% CI: 1.25–6.75; P: 0.013) false negative results (32 [25.2%]) compared to those having cleaning solution (9 [12.2%]). Laboratories having dirty smears had 2.65 (AOR: 2.65; 95% CI: 1.14–6.18; P: 0.024) times more false negative results (14 [36.8%]) compared to those laboratories with clean AFB smears (27 [16.6%]) (Table 3).

**Table 3:** Factors for false negative smear microscopy results in West Amhara region, 2014.

		False negative				
Variables		No	Yes	COR (95% CI)	AOR (95% CI)	P value
**Separate microscope**	No	136	31	0.55 (0.24–1.26)	0.45 (0.18–1.08)	0.075
	Yes	24	10	1	1	
**Lens cleaning solution**	No	95	32	2.43 (1.09–5.44)	2.90 (1.25–6.75)	0.013
	Yes	65	9	1	1	
**Filter 1% CF**	No	106	33	2.10 (0.91–4.86)	-	-
	Yes	54	8	1	-	-
**Normal size**	No	64	24	2.12 (1.01–4.25)	-	-
	Yes	96	17	1	-	-
**Even**	No	94	32	2.50 (1.12–5.58)	2.16 (0.91–5.11)	0.081
	Yes	66	9	1	1	
**Cleanness**	Dirty	24	14	2.94 (1.35–6.40)	2.65 (1.14–6.18)	0.024
	Clean	136	27	1	1	

AOR: Adjusted Odds Ratio; CI: Confidence Interval; COR: Crude Odds Ratio.

Similarly, no previous participation in EQA program was correlated with false positive results. Those laboratories with no EQA participation in 2013 had 3.43 (AOR: 3.43; 95% CI: 1. 39-8.45; P: 0.007) times more false positives (11 [25.0%]) than previously EQA linked laboratories (18 [11.5%]) (Table 4).

**Table 4:** Factors for false positive smear microscopy results in West Amhara region, 2014.

		False Positive				
Variables		No	Yes	COR (95% CI)	AOR (95% CI)	P value
**Filter paper**	No	75	17	1.83(0.83–4.07)	2.20 (0.94–5.16)	0.071
	Yes	97	12	1	1	
**Reagent labeling**	No	16	6	2.54(0.90–7.16)	2.74 (0.93–8.06)	0.067
	Yes	156	23	1	-	
**EQA Participated**	No	33	11	2.57(1.11–5.97)	3.43 (1.39–8.45)	0.007
	Yes	139	18	1	1	
**Normal size**	No	72	16	1.71(0.77–3.77)	-	
	Yes	100	13	1	-	
**Even**	No	103	23	2.57 (0.99–6.63)	-	
	Yes	69	6	1	-	
**Filter 1% CF**	No	177	22	1	-	
	Yes	55	7	1.48 (0.60–3.67)	-	

AOR: Adjusted Odds Ratio; CI: Confidence Interval; COR: Crude Odds Ratio; CF: Carbol Fuchsin; EQA: External Quality Assessment.

## Discussion

Reliable laboratory service provides results that are consistently accurate. Failure to maintain the quality would result in loss of credibility, waste of precious resources, inaccurate data and poor performance of the program [9]. In this study, 23.4% of laboratories had major errors. This is unacceptable performance and may indicate gross technical deficiencies [4]. Although this finding is lower compared to the 61.5% major errors found in democratic republic of Congo [8], it is higher compared to the only one major error (high false positive) reported in Afghanistan [10] and the 16.7% major errors reported in New Delhi [11]. The difference might be due to methodological differences that our study used blind rechecking method to check the routine performance of the laboratories, and others reports used panel testing method.

In the present study, false positive results were found in 14.4% of laboratories. These results could lead a patient placed on treatment unnecessarily, wasted valuable medication, cause emotional trauma to patients and their families [2, 4, 6]. The high false positive rate found in this study is higher compared to different studies conducted in Tanzania, India and Iraq that reported no false positive result [12–14]. These false positive results found in this study were significantly higher among EQA non participant laboratories before July 2013. This implies that EQA participant laboratories were more effective to reduce false positive results. Strengthened EQA program is crucial to implement standards uniformly, identify the root causes of the problems, suggest corrective measures and avoid the recurrence of the problems [8].

Low SPR and/ or ANSV are indicators of poor sputum smear microscopy performance. In this study, 83.1% of the laboratories had SPR of below 5%. Both low SPR and ANSV were found in 39.8% of laboratories. This finding is higher compared to the 2.9% of diagnostic centers with low SPR (< 5%) and low ANSV (< 301 slides) reported in New Delhi [8]. These microscopic centers should be intensively assessed to minimize false negative results or improper referral of TB suspects, and should be discontinued if they cannot be improved [6].

In this study, 41 (20.4%) laboratories had false negative results. These false negative results could cause the spread of TB to family and community, and death [6]. The finding is similar with a study done in Vietnam that reported 18.7% of false negative results [15]. However, this is higher compared to studies done in India and Iraq that reported the 3.7% and 2.3% of false negative results, respectively [13, 14]. False negative results may be due to dirty smear and dirty lenses caused by unavailability of microscope lens cleaning solution and poor smear cleanness.

The sensitivity and reliability of sputum smear microscopy often depend on how specimens are collected, smears are made and stained smears are examined [16]. This study has found poor specimen quality, smear thickness and evenness in 134 (66.7%), 133 (66.2%) and 126 (62.7%) laboratories, respectively. Similarly, more than half of AFB slides had smearing problems in Taiwan [17]. This performance is poor compared to the 80% smear quality cut off value set by the national guideline [6]. It is also poor compared to studies done in Argentina and Tanzania that reported acceptable specimen quality and well prepared smears [12, 18]. Such poor smears could cause false negative results [18, 19]. The absence of salivary specimen rejection due to HIV patients may affect the smears evenness, thickness and specimen quality.

This study was not without limitation since unable to include proficiency testing due to budget constraint.

## Conclusions

The performance of health centers for smear microscopy reading and smear quality were poor in west Amhara region. Unavailability of microscope lens cleaning solution and poor smear cleanness were factors for false negatives whereas no previous participation in EQA program for false positive results. Hence, strengthening the EQA program and technical support on smear quality indicators are recommended to ensure quality tuberculosis diagnostic service.
